# Microleakage and shear bond strength of orthodontc brackets bonded to hypomineralized enamel following different surface preparations

**DOI:** 10.4317/jced.51254

**Published:** 2014-04-01

**Authors:** Mostafa Shahabi, Farzaneh Ahrari, Hamideh Mohamadipour, Horieh Moosavi

**Affiliations:** 1 DDS, MS, Associate Professor of Orthodontics. Dental Research Center, School of Dentistry, Mashhad University of Medical Sciences, Mashhad, Iran; 2 DDS, MS, Assistant Professor of Orthodontics. Dental Research Center, School of Dentistry, Mashhad University of Medical Sciences, Mashhad, Iran; 3 DDS MS, Assistant Professor. Department of Operative Dentistry, School of Dentistry, Mashhad University of Medical Sciences, Mashhad, Iran; 4 DDS MS, Assistant Professor of Operative Dentistry. Dental Material Research Center, School of Dentistry, Mashhad University of Medical Sciences, Mashhad, Iran

## Abstract

Objectives: This study investigated the effects of several conditioning methods on shear bond strength (SBS) and microleakage of orthodontic brackets bonded to demineralized enamel. Study Design: One hundred premolars were selected and immersed in a cariogenic solution for 12 weeks. The teeth were randomly assigned into 5 groups. In groups 1 and 2, the teeth underwent acid etching for 30 and 120 seconds, respectively. In group 3, a combination of laser and acid etching was employed. A self-etch primer (SEP) was applied in group 4 and in group 5, the teeth were exposed to acidulated phosphate fluoride (APF) for 4 minutes before etching. After bracket bonding, the teeth were immersed in methylen blue for 12 hours and then were mounted in acrylic resin. SBS was determined with an Instron Universal Testing Machine and the amount of microleakage under the brackets was assessed under a stereomicroscope. Results: The lowest SBS was related to the SEP group and the highest one was observed in the specimens prepared by APF+acid etching. There was a significant difference in SBS (p=0.009), but not in microleakage (p=0.971) of the study groups. The SBS of the specimens treated with SEP was significantly Lower than the other groups, which were not significantly different from each other. The SEP group displayed a higher frequency of bond failure at the enamel-adhesive interface. Conclusions: Enamel preparation with SEP provided the lowest SBS among the groups. All groups showed some degree of microleakage. There was no significant correlation between SBS and microleakage.

** Key words:**Bond strength, microleakage, bonding, self-etch primer, Er:YAG laser.

## Introduction

The success of the bracket bonding procedure depends on the surface characteristics of the enamel. Sometimes, patients seeking orthodontic treatment show hypomineralized enamel because of environmental factors such as white spot lesions or due to a systemic condition called molar-incisor hypomineralization (MIH). MIH has been defined as hypomineralization of systemic origin in one to four permanent first molars, which is frequently accompanied with affected incisors (1). The teeth with hypomineralized defects usually show high failure rates of adhesives because the surface characteristics of the involved enamel prevent from achieving the etching patterns observed in sound enamel (2,3). A previous study found that brackets bonded to teeth with demineralized enamel indicated significantly lower bond strength compared to those attached to normal enamel (2). William *et al.* (3) reported that the enamel-adhesive interface in hypomineralized teeth was porous and with cracks, causing structural weakness. Therefore, searching for a supplementary surface preparation technique for reinforcing adhesion to hypomineralized enamel has been considered as an issue of interest.

Previous studies demonstrated the occurrence of microleakage under orthodontic brackets bonded to sound enamel (4-6). This has been attributed to the polymerization shrinkage of adhesive resins and also to the different rates of expansion and contraction of adhesive, teeth and brackets following exposure to the temperature changes in the mouth, which allows penetration of oral fluids and bacteria within the gap created at the enamel-adhesive-bracket complex (4,5). The risk of microleakage may be higher in patients with hypomineralized teeth because of the decreased adhesion to affected enamel.

Different techniques have been suggested to improve the bonding interface of hypomineralized enamel. Some reports extended the etching time over the 30 seconds in order to increase bond strength of adhesives to affected enamel (7,8). William *et al.* (3) demonstrated better bonding of a self-etch adhesive than a total-etch system to teeth with molar incisor hypomineralization. Laser etching is another method of surface conditioning that can provide microporosities on the surface (9,10), and in combination with acid etching, it can provide bond strength values that are comparable or even higher than the conventional acid etching technique (11,12). It has also been demonstrated that fluoride treatment followed by acid etching of enamel caries or hypomineralized enamel can produce etching patterns similar to those observed in etched sound enamel, while restoring the mineral lost during lesion formation (13,14).

There are controversial reports regarding the relationship between microleakage and bond strength. In theory, the quality and strength of the bond should prevent from penetration of fluids and bacteria under orthodontic brackets and thus decrease microleakage, but such a relationship has not been confirmed by some authors (6). The purpose of the present study was to investigate the effects of several surface preparation methods on shear bond strength (SBS), mode of bond failure and microleakage of brackets bonded to teeth with demineralized enamel and to assess any significant relationship between SBS and microleakage.

## Material and Methods

One hundred premolar teeth extracted for orthodontic reasons were gathered and stored in saline solution until the time of the experiment. Teeth with caries, cracks, and developmental defects were excluded from the sample. All the teeth were immersed for 12 weeks in a cariogenic solution consisting of 2.2 mM CaCl2, 2.2 mM NaH2PO4, and 50 mM acetic acid (PH 4.8) to create demineralized enamel (15). This solution was replaced weekly. After that, the demineralized teeth were rinsed with tap water and randomly divided into five groups of 20 each. The buccal surfaces of the teeth in the experimental groups were cleaned with water slurry of pumice and rubber prophylactic cups for 5 seconds and then underwent the following surface preparation procedures:

Group 1 (control): The buccal surfaces of the demineralized teeth were covered with a 37% phosphoric acid gel for 30 seconds, thoroughly rinsed with water and dried with an oil-free air source. The chalky-white appearance was observed after drying.

Group 2: The enamel surfaces were exposed to a 37% acid phosphoric gel which was applied for an extended period of 2 minutes. The teeth were then rinsed with a copious amount of water and air-dried.

Group 3: An Er:YAG laser (wavelength 2940 nm; Smart 2940 D, Deka Laser, Firenze, Italy) was used for conditioning the enamel surface before the conventional acid etching process. The laser operated at 10 Hz, 150 mJ of energy, very short pulse and the bonding area was etched for 10 seconds using scanning movements. Procedures after laser conditioning were the same as that used in the control group.

Group 4: A mild self-etch primer (Clearfil SE Bond, Kuraray Co. Ltd., Osaka, Japan) was applied for enamel preparation, according to the manufacturer’s recommendations.

Group 5: The demineralized enamel was covered with a sufficient thickness of acidulated phosphate fluoride gel (Sultan Healthcare Inc., Englewood, New Jersey, USA) for 4 minutes, and then was rinsed for two consecutive periods of 5 minutes each to remove any readily-soluble reaction products (14). Conventional acid etching was later applied similar to the control group.

After surface preparation, a thin coat of Transbond XT primer (3M Unitek, Monrovia, California, USA) was painted on the enamel surface. Stainless steel pre-adjusted edgewise upper premolar brackets (0.022-inch slot; Dentsply GAC International, Bohemia, NY, USA) with an average base area of 13.1 mm2 were used in this study. Each bracket was firmly pressed at the middle of the clinical crown using Transbond XT adhesive (3M Unitek), and the excess material was removed from around the base with a sharp explorer. The adhesive was polymerized from each of the occlusal, gingival, mesial and distal directions (10 seconds each) for a total of 40 seconds using a light-emitting diode (LED) device (Bluephase C8; Ivoclar Vivadent, Schaan, Liechtenstein) at power density of 650 mW/cm2.

After bonding, the specimens were stored in distilled water for at least 48 hours at 37oC and then subjected to a thermocycling process which was performed between 5ºC to 55ºC for 500 cycles, with dwell time of 30 seconds per bath. Microleakage was assessed by the dye penetration technique. For this purpose, the teeth apices were sealed with sticky wax and the entire surfaces of the teeth were covered by two consecutive layers of nail varnish up to 1 mm around the bracket margins. The specimens were then immersed in a solution of 1 per cent methylene blue dye for 12 hours at room temperature. After removing from the solution, the teeth were tho-roughly rinsed, and then were mounted in plastic rings poured with self-curing acrylic resin, so that the buccal surfaces of the teeth were parallel to the direction of the debonding force.

Shear bond strength (SBS) testing was performed in an Instron Universal Testing machine (Santam, model STM-20, Iran) using cross head speed of 1 mm per minute until failure. The force required to detach the bond was recorded in newtons and then was divided by the surface area of the bracket base to provide bond strength value in megapascals (N/mm2).

After debonding, the teeth were examined by one calibrated examiner under a stereomicroscope (Dino-Lite Pro, Anmo Electronics Corp, Taiwan) at 20X magnification and the deepest penetration of the dye under the brackets was measured in millimeter perpendicular to the bracket margin. To examine the measurement error, 20% of the specimens were randomly selected and re-examined with an interval of one week. The measurement error was determined using the Dahlberg formula and the systemic error of the two measurements was assessed by the Wilcoxon signed rank test.

The bonding interface was also examined with the stereomicroscope at 10X magnification to assess the mode of bond failure. This was determined on the basis of the amount of adhesive adhered to the enamel surface according to the adhesive remnant index (ARI) of Artun and Bergland (16):

Score 0: no adhesive remained on the tooth, Score 1: less than 50% of the adhesive remained on the tooth, Score 2: more than 50% of the adhesive was left on the tooth, Score 3: the entire adhesive remained on the tooth with distinct impression of the bracket base.

- Statistical analysis

The normality of the SBS data was confirmed by the Kolmogorov-Smirnov test. One way analysis of variance was used to determine any significant difference in SBS values among the study groups, followed by Tukey multiple range test for pairwise comparisons. The ARI scores were analyzed with Fisher’s exact test and the difference in microleakage values of the study groups was detected by Kruskal-Wallis test. Spearman’s rank correlation test was used to assess any significant relationship between bond strength and microleakage. The data were analyzed by SPSS (Statistical Package for Social Sciences, Version 16.0, Chicago, Illinois, USA) software and the level of significance was predetermined at p<0.05.

## Results

The intra-examiner systemic error was not significant between the two microleakage assessments (*p*=0.217) with a measurement error of 0.37 mm.

 Table 1 displays the mean, standard deviation (SD) and range of the SBS values (MPa) and the results of statistical analysis for between-group comparison. The lowest SBS was related to the specimens prepared with the SEP and the highest one belonged to those treated with APF before conditioning. ANOVA displayed a significant difference in bond strength of the study groups (*p*=0.009). Multiple comparisons by Turkey test revealed that the SBS of the specimens treated with the SEP was significantly lower than the other study groups, which were not significantly different from each other ( Table 1).

**Table 1:**
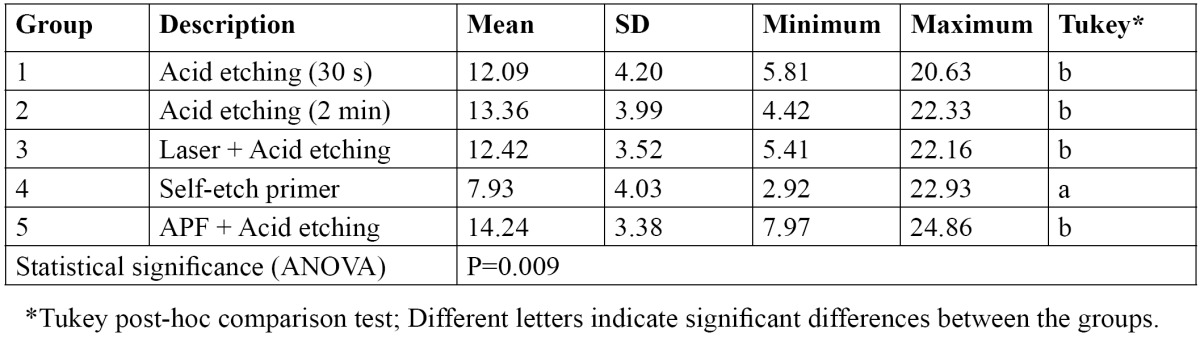
Descriptive statistics and the results of statistical analysis for comparison of SBS values (MPa) among the study groups.

The results for the ARI scores are demonstrated in  Table 2. The Fisher’s exact test exhibited a significant difference in the distribution of ARI scores among the study groups (p<0.001). The specimens in the SEP group showed a higher frequency of bond failure at the enamel-adhesive interface (no adhesive remained on the enamel) compared to the other study groups.

**Table 2:**
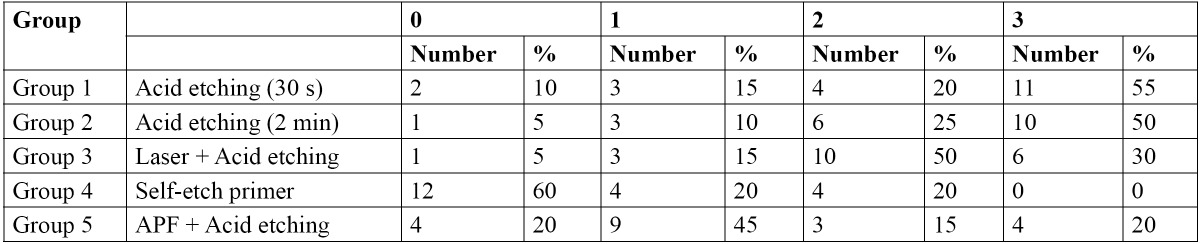
The distribution of ARI scores in the study groups.

**Figure 1:**
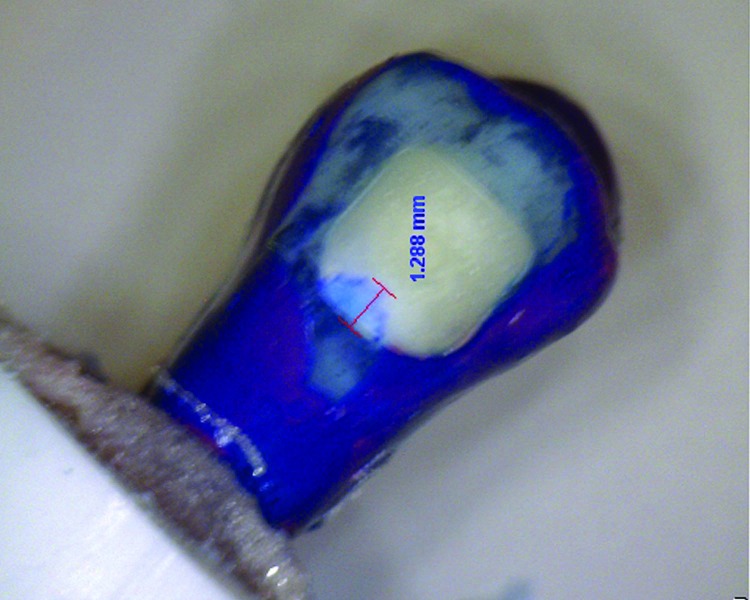
The occurrence of microleakage under a bracket.

 Table 3 indicates the descriptive statistics relating to the microleakage values of the study groups. All groups displayed some degree of microleakage beneath the brackets. An example of microleakage has been illustrated in figure 1. The statistical comparison by Kruskal-Wallis test exhibited no significant difference in microleakage among the study groups ( Table 3).

**Table 3:**
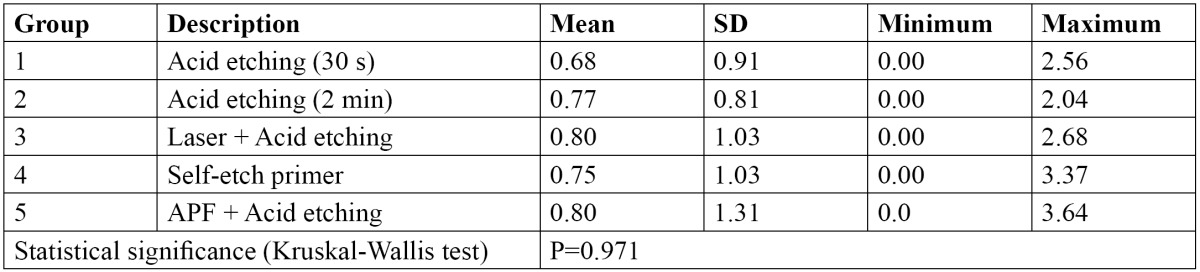
Descriptive statistics and the results of statistical analysis for comparison of microleakage values (mm) among the study groups.

The Spearman’s rank correlation coefficient revealed no significant relationship between bond strength and microleakage (r=-0.018, *P*=0.749).

## Discussion

The present study investigated some methods to improve bond strength of brackets bonded to caries-like lesions of enamel. The bond strength of demineralized teeth treated with conventional acid etching was lower than those obtained after 2 minutes acid etching, laser + acid etching or APF treatment followed by acid etching, although the differences were not statistically significant. Laser etching followed by acid etching increased the bond strength of orthodontic brackets to some degree, but the benefit was negligible. We used laser etching in combination with acid etching because the efficacy of laser etching alone for enamel conditioning may be lower than the conventional acid etching technique. Laser etching can have benefits in reducing decalcification and future caries attack resulted from preparing enamel with phosphoric acid (17-19), although its caries-resistant benefit has been questioned by some authors (20).

The increased etching time can be a useful strategy to provide enough adhesion in teeth with enamel defects. In the present study, after increasing the etching time to 2 minutes, the bond strength improved from 12.09 MPa to 13.36 MPa, but the difference was not statistically significant. Therefore, the benefit achieved from increased etching time in teeth with demineralized enamel is little, but this technique may be suitable in cases with other physicochemical alterations in the enamel structure. The disadvantages are the increased working time, which may be burdensome for the clinician as well as for the patients, and the risk of increased surface loss and further decalcification of enamel around orthodontic attachments.

Self etch primers (SEP) are being popular in orthodontic treatments because they reduce chair time and simplify the bonding technique. Considering that the demineralized teeth show lower mineral content than the sound enamel, creating a more conservative etching pattern and less enamel demineralization by using a SEP may be advantageous. William *et al.* (3) reported that the SBS of composite to hypomineralized enamel was higher when a self-etch adhesive was used instead of a total-etch system. They proposed that SEP prevents from water trap in the porous lattice of hypomineralized enamel by omitting the rinsing step, and it may also provide chemical adhesion in addition to the micromechanical retention to hydroxyapatite (3). In this study we used a mild self-etch primer commonly used in restorative treatments and although the resultant bond strength was significantly lower than that of the other study groups, it was considered clinically acceptable.

In the present study, fluoride treatment followed by acid etching of demineraized enamel produced the highest SBS in teeth with demineralized enamel. Previous studies found that fluoride treatment followed by acid etching of caries-like lesions provided etching patterns that were suitable for adhesive placement, while creating a rapid supply of fluoride for remineralization (13,14). Although fluoride therapy prior to acid etching of demineralized enamel enhanced the bond strength from 12.09 to 14.24 MPa, but this increase was not statistically significant. This is in contrast to the findings of Shahabi *et al.* (2) who found that application of 2% sodium fluoride (NaF) prior to acid etching of demineralized enamel caused a significant increase in bond strength of orthodontic brackets. This controversy may be related to the application of APF gel in this steady instead of a neutral fluoride agent in the study of Shahabi *et al.* (2). The findings of the present study advocate the use of fluoride agents before the bonding procedure in cases of enamel caries in order to reharden the enamel and create a hypermineralized surface layer which would be more resistant to caries progression (14). This may have great clinical implications when one considers the high prevalence (21) and the rapid development (22) of enamel caries in patients undergoing orthodontic treatment.

In the current study, all groups showed some degree of microleakage under orthodontic brackets with amounts ranging from 0.68 mm to 0.80 mm. Microleakage may result in clinical consequences such as formation of white spot lesions, enamel discoloration, corrosion and decreased bond strength of brackets. Microleakage under brackets bonded to demineralized enamel can aggravate the caries process initiated beforehand. A previous study found that microleakage at the enamel-adhesive interface was greater in demineralized teeth compared to those with sound enamel, although the difference was not significant (23). In this study, the lowest microleakage value (0.68 mm) was observed in the standard acid etching group and the highest one (0.80 mm) occurred in the laser + acid etching and APF + acid etching groups. However, the differences between groups were small and not statistically significant, indicating that none of the surface preparation techniques were able to cause a significant decrease in microleakage. This is in contrast to the findings of Moosavi *et al.* (23) who found that microleakage was significantly reduced after NaF treatment followed by acid etching of demineralized enamel. However, the method of sample preparation for microleakage assessment and the type of fluoride agents they employed were different from those used in this study. The outcomes of this study are also in contrast with those of Hamamci *et al.* (24) who reported that enamel preparation with Er:YAG and Er,Cr:YSGG lasers resulted in significantly higher microleakage than when the standard acid-etching was used. Several authors demonstrated significantly higher microleakage after application of a self etch primer for bonding brackets (23-25), but the results of this study using Clearfil SE Bond did not agree with these authors.

The present study did not report a significant relationship between SBS and microleakage. This may be because the SBS and microleakage values of the most study groups were close to each other and a wide range of values was not observed either in SBS or in microleakage. Although application of a self-etch primer caused a significant decrease in SBS, the microleakage value of this group was not statistically different from the other groups, indicating that microleakage is also influenced by other factors than only the adequacy of adhesion to enamel structure. The findings of this study corroborate the results of James et al. (6) who demonstrated no significant correlation between SBS and microleakage of orthodontic brackets bonded with various adhesives and curing systems. In contrast, Abdelnaby *et al.* (26) found a significant reverse relationship between SBS and microleakage, although the correlation coefficient was relatively low (r=-0.318).

The ARI scores provide some insight into the bond failure interface (27). In the present study, the ARI scores were significantly different among the study groups, indicating the differences in the distribution of bond failure interface. There was a higher percentage of ARI score 0 (bond failure at the enamel-adhesive interface) in the specimens prepared by SEP compared to the other study groups. This can be ascribed to the inadequate adhesion of composite to enamel prepared by by the SEP, as represented by the significantly lower bond strength. The bond failure in other study groups occurred mainly at the bracket-adhesive interface or within the adhesive itself.

In the present study, the SBS values of the study groups were higher than the 6-7.8 MPa, which is considered as the minimum SBS required in orthodontic clinical practice (28). The occurrence of microleakage although was small but warrants further attention to take measures to prevent enamel caries under orthodontic brackets. It should be noted that intraoral environment is different from laboratory conditions, so further clinical studies are suggested in this field.

## Conclusions

Some degree of microleakage occurred beneath brackets in all groups, with no significant difference to each other.

Conditioning of demineralized enamel with phosphoric acid for 120 seconds had no significant effect on SBS and microleakage of brackets bonded to demineralized enamel.

Laser etching in combination with standard acid etching neither influenced the SBS nor microleakage of brackets bonded to demineralized teeth.

Application of acidulated phosphate fluoride for 4 min before acid etching of demineralized enamel produced the highest SBS among the study groups, but the difference with most of the other groups was not statistically significant. Microleakage beneath the brackets was not affected by this procedure.

The SBS of brackets prepared with a self-etch primer was significantly lower than the other groups. The bond failure occurred predominantly at the enamel-adhesive interface.

There was no significant correlation between SBS and microleakage.
